# Ultrasound-aided Multi-parametric Photoacoustic Microscopy of the Mouse Brain

**DOI:** 10.1038/srep18775

**Published:** 2015-12-21

**Authors:** Bo Ning, Naidi Sun, Rui Cao, Ruimin Chen, K. Kirk Shung, John A. Hossack, Jin-Moo Lee, Qifa Zhou, Song Hu

**Affiliations:** 1 Department of Biomedical Engineering, University of Virginia, Charlottesville, VA 22908; 2 Department of Biomedical Engineering, University of Southern California, Los Angeles, CA 90089; 3 Department of Neurology, Washington University School of Medicine, St. Louis, MO 63110.

## Abstract

High-resolution quantitative imaging of cerebral oxygen metabolism in mice is crucial for understanding brain functions and formulating new strategies to treat neurological disorders, but remains a challenge. Here, we report on our newly developed ultrasound-aided multi-parametric photoacoustic microscopy (PAM), which enables simultaneous quantification of the total concentration of hemoglobin (C_Hb_), the oxygen saturation of hemoglobin (sO_2_), and cerebral blood flow (CBF) at the microscopic level and through the intact mouse skull. The three-dimensional skull and vascular anatomies delineated by the dual-contrast (i.e., ultrasonic and photoacoustic) system provide important guidance for dynamically focused contour scan and vessel orientation-dependent correction of CBF, respectively. Moreover, bi-directional raster scan allows determining the direction of blood flow in individual vessels. Capable of imaging all three hemodynamic parameters at the same spatiotemporal scale, our ultrasound-aided PAM fills a critical gap in preclinical neuroimaging and lays the foundation for high-resolution mapping of the cerebral metabolic rate of oxygen (CMRO_2_)—a quantitative index of cerebral oxygen metabolism. This technical innovation is expected to shed new light on the mechanism and treatment of a broad spectrum of neurological disorders, including Alzheimer’s disease and ischemic stroke.

The brain accounts for more than 20% of our oxygen consumption at the resting state^1^. Disruptions in cerebral oxygen metabolism play a key role in the initiation and progression of multiple life-threatening brain disorders, in particular Alzheimer’s disease and ischemic stroke^2^,^3^. High-resolution imaging of the cerebral metabolic rate of oxygen (CMRO_2_) in mice—a species with abundant disease models and genetic manipulations available—is crucial for understanding elusive pathogenic mechanisms and formulating new therapeutic strategies. However, existing techniques have yet to achieve this goal. Positron emission tomography (PET) allows quantifying CMRO_2_ in absolute values, but lacks the spatial resolution to image the mouse brain^4^,^5^. Combining optical intrinsic signal and laser speckle imaging allows measuring CMRO_2_ at the mesoscopic level, but rather qualitative^6^,^7^. Functional ultrasound enables high resolution imaging of the cerebral blood flow across the entire rodent brain^8^,^9^, but does not have access to the functional information of blood oxygenation.

Photoacoustic microscopy (PAM)^10^,^11^,^12^ holds great potential to address this long-standing challenge. Capitalizing on the optical absorption of hemoglobin—the primary carrier of oxygen in the circulation, PAM allows *in vivo* characterization of vascular anatomy^13^,^14^, hemodynamics^15^, and vasoactivity^16^,^17^,^18^. By measuring the total concentration of hemoglobin (C_Hb_), the oxygen saturation of hemoglobin (sO_2_), and blood flow at selected locations in feeding arteries and draining veins, Yao *et al.* previously demonstrated PAM of the total metabolic rate of oxygen in the tumor-bearing mouse ear^19^ and recently extended it to measure relative CMRO_2_ changes in the electrically stimulated mouse brain^20^. Although encouraging, this method is not readily applicable for high-resolution CMRO_2_ imaging, because the three parameters were not simultaneously quantified and the cerebral blood flow (CBF) was not measured at the same spatial scale as the other two parameters.

To fill the gap, we have developed an ultrasound-aided multi-parametric PAM platform, which is capable of imaging C_Hb_, sO_2_, and CBF at the same spatiotemporal scale. With the ultrasonically extracted contour map of the mouse skull, our PAM can dynamically focus on the underlying cortical vasculature when scanning across the uneven brain surface to maintain high spatial resolution and sensitivity. Statistical, spectral, and correlation analysis of the same PAM dataset allows simultaneous quantification of C_Hb_, sO_2_, and CBF at the microvascular level. Taking advantage of bi-directional raster scan, our PAM can further determine the direction of blood flow in individual vessels. With the future development of complementary algorithms to extend the three parameters to the tissue level, the ultrasound-aided multi-parametric PAM will ultimately enable us to derive microscopic CMRO_2_ using the Fick’s law.

## Results

### Mechanism of simultaneous multi-parametric PAM

**Figure 1:**
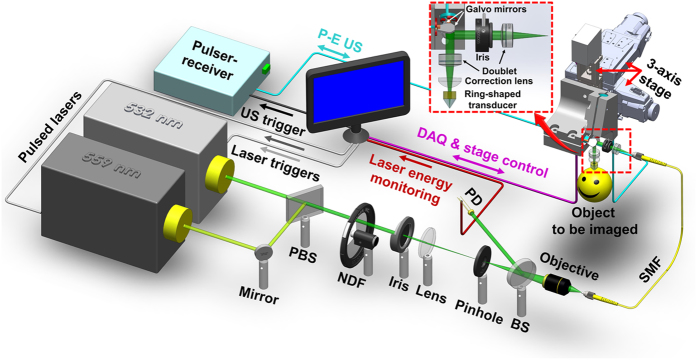
Schematic of the ultrasound-aided multi-parametric PAM. DAQ, data acquisition. P–E: pulse-echo. US: ultrasound. Figure prepared by Dr. Bo Ning.

Our PAM utilizes two nanosecond-pulsed lasers (wavelengths: 532 and 559 nm). Simultaneous high-resolution imaging of C_Hb_, sO_2_, and CBF is achieved through statistical, spectral, and correlation analysis of the dual-wavelength measurement. Specifically, PAM is insensitive to sO_2_ at 532 nm, a near-isosbestic point of hemoglobin where the optical absorption coefficients of oxy- and deoxy-hemoglobin (HbO_2_ and HbR, respectively) are equal. Fluctuations in the PAM signal acquired at 532 nm encode both the Brownian motion and the flow of red blood cells (RBCs)^21^. The Brownian motion-induced statistical fluctuation of the photoacoustic amplitude is independent of the blood flow speed and reveals the number of RBCs within the detection volume of PAM^22^, which can be used to compute C_Hb_ in absolute values. In parallel, the decorrelation rate of successively acquired A-line signals is proportional to the speed of blood flow^23^, which can be combined with the vessel diameter to derive CBF in volumetric units. Combining the readouts at both wavelengths, PAM can differentiate HbO_2_ and HbR based on their distinct absorption spectra to quantify sO_2_ in absolute values^24^. See Methods for details about the experimental setup (Fig. 1) and procedures for the quantification of C_Hb_, sO_2_, and CBF.

### System performance

**Figure 2:**
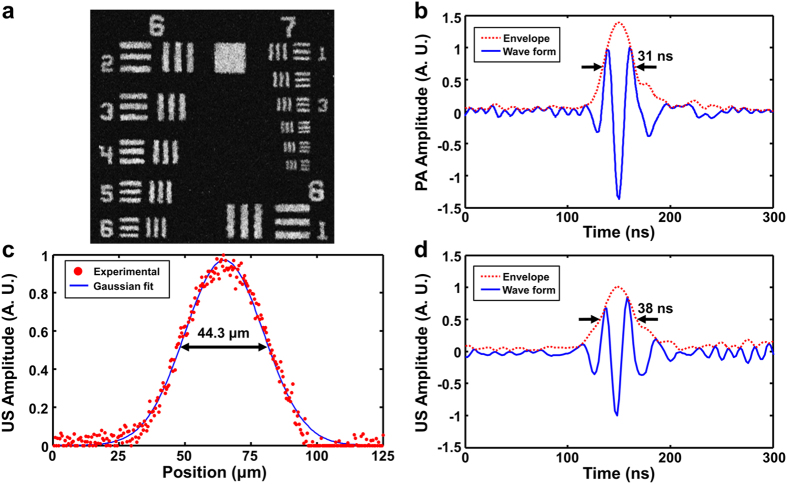
Performance of the ultrasound-aided multi-parametric PAM. (**a**) Lateral resolution of PAM quantified using a resolution target. (**b**) Axial resolution of PAM quantified by the FWHM value of the A-line photoacoustic envelope of a 7-μm carbon fiber. (**c**) Lateral resolution of SAM quantified by the FWHM value of the Gaussian-fitted cross-sectional profile of the same carbon fiber. (**d**) Axial resolution of SAM quantified by the FWHM value of the A-line ultrasonic envelope of the carbon fiber.

The optically defined lateral resolution of PAM was characterized using a resolution target (R1DS1P, Thorlabs). As shown in Fig. 2a, our platform clearly resolved the 6^th^ element of Group 7. By fitting the experimentally measured modulation transfer function (MTF) to the theoretical MTF of a “perfect” optical system^25^, we estimated the cutoff spatial frequency to be 365.4 line pair/mm, corresponding to a lateral resolution of 2.7 μm. Although slightly worse than the diffraction-limited resolution (2.0 μm), it is sufficient to resolve single capillaries. The acoustically defined axial resolution of PAM was estimated by imaging a 7-μm carbon fiber (S-CF706-T700, CST), whose diameter is much smaller than the acoustic wavelength and thus serves as an “ideal” line target. The full-width at half-maximum (FWHM) value of the temporal envelope of the A-line photoacoustic signal was measured to be 31 ns (Fig. 2b), corresponding to an axial resolution of 46.4 μm (theoretically 38.0 μm).

The resolution of the integrated scanning acoustic microscopy (SAM) was characterized using the same carbon fiber. Laterally, the acoustically measured cross-sectional profile of the fiber showed a FWHM value of 44.3 μm (Fig. 2c), agreeing with the diffraction-limited acoustic focus (44.0 μm). Axially, the FWHM value of the temporal envelope of the A-line ultrasonic signal was measured to be 38 ns (Fig. 2d), corresponding to an axial resolution of 56.9 μm (theoretically 53.8 μm).

### Ultrasound-aided contour PAM: phantom and *in vivo* tests

To maintain high resolution and sensitivity for accurate quantification of C_Hb_, sO_2_, and CBF in the uneven mouse brain, we have expanded the contour PAM technique that was recently developed to address the out-of-focus issue in imaging the tumor-bearing mouse ear^26^. Following the surface contour of the tumor outlined by its densely packed vasculature in a pre-scanned photoacoustic image, the contour PAM can dynamically adjust the focal plane to accommodate the uneven tumor surface. Although encouraging, this technique is not directly applicable to the mouse brain, because skull vessels could be easily misidentified as cortical vessels and adversely influence the detection of the cortical contour. To address this challenge, we have integrated SAM in our platform. Compared with the previous implementation^26^, our ultrasound-aided contour PAM relies on direct pulse-echo imaging of the skull-cortex interface rather than inaccurate interpolation based on discretely distributed brain vessels. Moreover, the much larger depth of focus of SAM allows more reliable detection of the surface contour of the dome-shaped mouse cortex, which spans several hundred microns along the depth direction.

**Figure 3:**
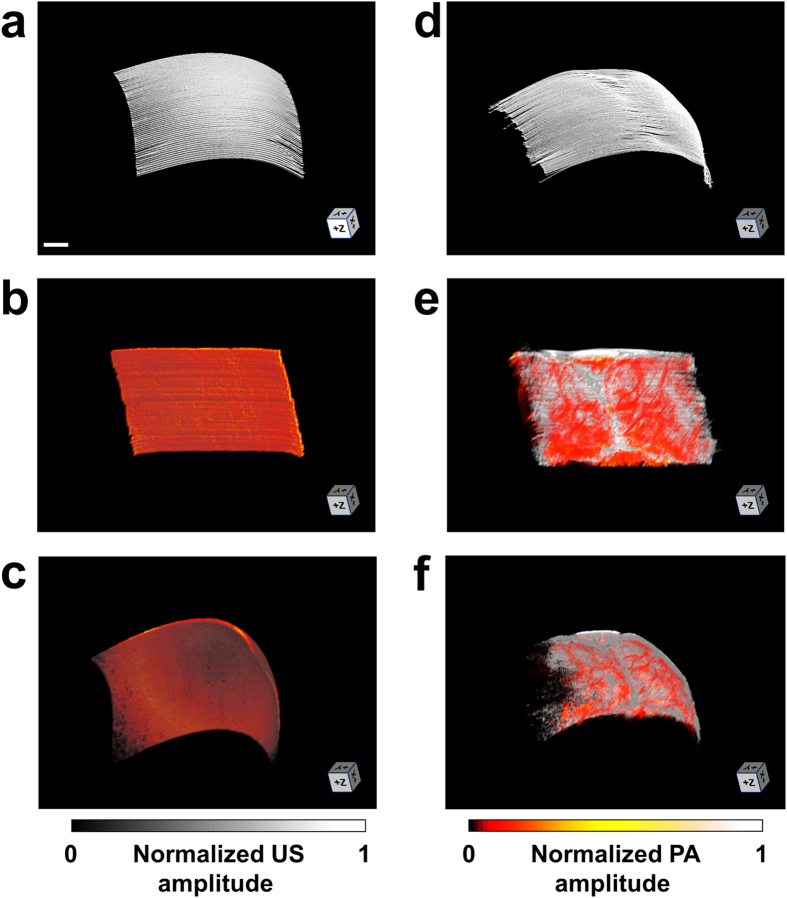
Phantom and *in vivo* tests of the ultrasound-aided contour scan. (**a**) Screenshot of the 3D surface contour of the plastic ball extracted by SAM. (**b**,**c**) Screenshots of the 3D rendering of the surface of the ball imaged by PAM with and without the contour scan, respectively. (**d**) Screenshot of the 3D surface contour of the mouse skull extracted by SAM. (**e**,**f**) Screenshots of the 3D rendering of the mouse brain simultaneously imaged by SAM (gray) and PAM (hot) with and without the contour scan, respectively. Scale bar: 1 mm.

We tested the performance of our ultrasound-aided contour scan using a plastic ball coated with black ink (diameter: 20 mm). First, as shown in Fig. 3a [see Supplementary Fig. S1 for three-dimensional (3D) visualization], the surface contour of the ball was extract by a rapid SAM scan (See Methods for details about the contour extraction). Then, a pair of PAM images with (Fig. 3b and Supplementary Fig. S2) and without (Fig. 3c and Supplementary Fig. S3) ultrasound-aided contour scan were acquired for comparison. Visibly, the sphere-shaped ball surface shown in the conventional PAM image became flat in the contour image, due to the dynamically adjusted focal plane. Moreover, the top surface and lower periphery of the ball—which were out of focus and thus dim and fuzzy in the conventional PAM image—became bright and clear in the contour image, indicating the improvement in both sensitivity and spatial resolution.

Following the phantom study, we further tested the system performance *in vivo*. Similarly, a 6 × 8 mm^2^ region of the mouse brain was imaged by SAM to map the skull contour (Fig. 3d and Supplementary Fig. S4). Then, the same region of interest was imaged by our dual-contrast platform with and without the contour guidance. Since PAM and SAM shared the same acoustic detection, the concurrently acquired photoacoustic and ultrasonic images were automatically co-registered and readily fusible. As shown in Fig. 3e (see Supplementary Fig. S5 for 3D visualization), the entire mouse skull (imaged by SAM) and underlying cortical vasculature (image by PAM) are visually flat, due to the contour-guided dynamic focusing. Better maintaining the spatial resolution and sensitivity, the contour scan clearly resolved the microvasculature near the junction of parietal and temporal cortices, which were out of the focal plane of conventional PAM (Fig. 3f and Supplementary Fig. S6).

### Multi-parametric transcranial PAM of the mouse brain

**Figure 4:**
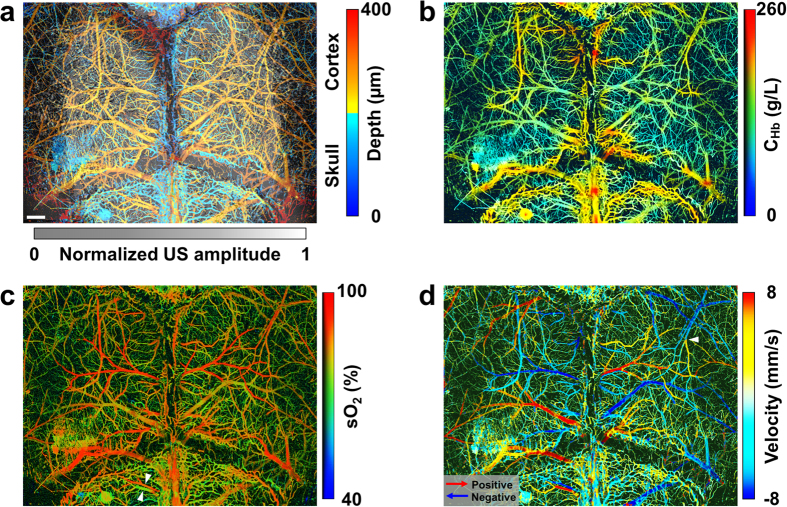
Ultrasound-aided multi-parametric PAM of the mouse brain through the intact skull. (**a**) Depth-encoded skull vasculature (labeled in cold color) and cortical vasculature (in warm color) separated by the SAM-determined skull (in gray). US: ultrasound. (**b**–**d**) Simultaneously acquired high-resolution maps of C_Hb_, sO_2_, and CBF (both speed and direction), respectively. The arrows in panel (**c**) indicate a pair of cortical arteriole and venule identified by their distinct sO_2_ values. The red and blue arrows in panel (**d**) indicate the directions of the blood flow along the B-scan axis, and the white arrow indicates an arteriole whose orientation is nearly orthogonal to the B-scan axis. Scale bar: 0.5 mm.

Capitalizing on the high spatial resolution and wide field of view of the ultrasound-aided contour PAM, we demonstrated—for the first time—simultaneous transcranial mapping of C_Hb_, sO_2_, and CBF over the entire mouse cortex. Relying on the 3D skull anatomy acquired by SAM, the vascular networks in the skull and underlying cortex were clearly separated (Fig. 4a). Note that the depth range (up to 400 μm) does not reflect the maximum penetration of PAM, because the maximum rather than the deepest signal is projected along each A-line.

The C_Hb_ was quantified in absolute values by statistical analysis of the Brownian motion of RBCs in 100 successive A-lines acquired by PAM at 532 nm (see Methods for details). As shown in Fig. 4b, the average C_Hb_ was measured to be 113.7 ± 34.7 g/L, which was in agreement with the reported value^27^. Interestingly, the average C_Hb_ value in the skull (136.1 ± 28.7 g/L) was slightly higher than that in the cortex (100.1 ± 20.8 g/L). By analyzing the 100 dual-wavelength A-line pairs acquired at 532 and 559 nm, we computed the proportions of HbO_2_ and HbR in C_Hb_, from which sO_2_ was derived (Fig. 4c). The methodology for spectroscopic PAM of sO_2_ has been established and described before^24^. As a testament to its robust performance, PAM clearly identified a pair of cortical arteriole and venule partially shadowed by the microvessels in the interparietal skull (indicated by the white arrows in Fig. 4c). Relying on the RBC flow-induced decorrelation between the same 100 A-lines acquired at 532 nm, PAM quantified the speed of the blood flow in individual vessels (Fig. 4d). This method has been validated and utilized by us in the mouse ear^23^. It is worth noting that the blood flow in the mouse brain has both transverse and axial components, which is in contrast to the ear where blood circulates within the transverse plane (i.e., perpendicular to the imaging head). In light of this, we quantified the angle between the vessel axis and the transverse plane to derive the total CBF from its transverse component measured by the correlation analysis (see Methods for details). Taking into consideration the relative movement between the cross-sectional scanning (i.e., B-scan) stage and RBC, PAM further determined the CBF direction by capturing the subtle difference in the relative flow speeds measured using forward and backward B-scans. This method has been previously utilized for flow imaging in the mouse ear^28^. As shown in Fig. 4d, PAM can accurately trace the direction of blood flow in individual vessels (indicated by warm and cold colors), which nicely corresponds to the sO_2_ (i.e., the arterial blood flows from parent to daughter branches, while the venous blood flows oppositely). Strikingly, our PAM was able to pinpoint the direction of the blood flow in an arteriole, whose orientation was nearly orthogonal to the B-scan axis. As indicated by the white arrow in Fig. 4d, the two daughter branches bifurcated from the arteriole show different colors, indicating that they were flowing toward opposite directions along the B-scan axis but both away from the parent branch.

## Discussion

Relying on the capillary-level spatial resolution and considerable resistance to the skull-induced optical aberration, PAM holds great potential for transcranial imaging of CMRO_2_ at the microscopic level in mice, which provide ideal experimental settings for mechanistic studies of neurological disorders. To this end, we have made the important first step by developing the ultrasound-aided multi-parametric PAM for simultaneous imaging of C_Hb_, sO_2_, and CBF—the three parameters required to derive CMRO_2_. The future development of complementary algorithms to extended the PAM-measured parameters from the vascular level to the tissue level will ultimately prepare us to compute microscopic CMRO_2_ using the Fick’s law. A dynamic view of the co-development of metabolic dysfunction and neuronal damage revealed by PAM may revolutionize our current understanding of many neurological diseases and shed new light on neuroscience research.

## Methods

### Experimental setup

Our ultrasound-aided multi-parametric PAM platform (Fig. 1) employs two nanosecond-pulsed lasers (Edgewave, BX40-2-G and BX40-2-GR; wavelengths: 532 and 559 nm; repetition rate: 30 kHz) for dual-wavelength photoacoustic excitation. The two beams with orthogonal polarizations are combined through a broadband polarizing beamsplitter (PBS; Edmund Optics, 48–545), attenuated by a neutral-density filter (NDF; Thorlabs, NDC-50 C-2M), and reduced to the same diameter by an iris (Thorlabs, SM1D12D) for fiber-optic coupling. To enhance the coupling efficiency, the dual-color beam is focused by a condenser lens (Thorlabs, LA1608) and spatially filtered by a 50-μm-diameter pinhole (Thorlabs, P50C), before being coupled into a single-mode fiber (SMF; Thorlabs, P1-460B-FC-2) through a microscope objective (Newport, M-10X). To compensate for the fluctuation in laser intensity, ~5% of laser energy is tapped off by a beam sampler (BS; Thorlabs, BSF10-A) and monitored by a photodiode (PD; Thorlabs, FDS100).

As shown in the blow up of the imaging head (boxed region in Fig. 1), the near diffraction-limited fiber output is mapped into the object to be imaged by two identical doublets (Thorlabs, AC127-025-A) through an iris (Thorlabs, SM05D5), a two-axis galvo scanner (Cambridge, 6215HSM40B), a correction lens (Thorlabs, LA1207-A), and a home-made ring-shaped ultrasonic transducer (inner diameter: 2.2 mm; outer diameter: 4.0 mm; focal length: 6.0 mm; center frequency: 35 MHz; 6-dB intensity bandwidth: 70%). The LabVIEW-controlled galvo scanner can steer the laser beam through the central opening of the transducer for automated confocal alignment of the optical-acoustic dual foci. The iris is utilized to reduce the beam diameter to the dimension of the galvo mirrors, and the correction lens is used to compensate for the optical aberration at the interface between the ambient air and ultrasound-coupling liquid (water in our system). For the contour scan, the imaging head is motorized by a three-axis scanner, which consists of two transverse stages (PI miCos GmbH, PLS-85) for raster scan and one vertical stage (THK, KR15; motor: Circuit Specialists, 28BYG201) for dynamic adjustment of the focal plane. A pulser-receiver (Olympus, 5900PR) is utilized to drive the ultrasonic transducer for SAM.

### Procedure for ultrasound-aided contour extraction

The three-step procedure begins with a rapid ultrasonic scan of the region of interest. The B-scan speed and the repetition rate of the pulser-receiver are set to 5 mm/s and 1 kHz, respectively. With a relatively large scanning interval between adjacent B-scans (40 μm), which is comparable to the lateral resolution of SAM, it takes only 4 minutes to image a 6 × 8 mm^2^ region. Then, a self-developed MATLAB program is applied to identify the depth of the maximum signal in each A-line. Integrating the depth information extracted from individual A-lines leads to a 3D map of the surface contour. Finally, the ultrasonically extracted contour map is interpolated (down to the same step sizes planned for the contour scan) and smoothed (with a span of 15% of the B-scan length) for the contour-guided multi-parametric PAM.

### Scanning scheme of ultrasound-aided multi-parametric PAM

We have designed a novel scanning scheme to simultaneously acquire the hemodynamic and anatomical information of the mouse brain. Specifically, the B-scan speed is set to 1 mm/s, during which the two lasers are alternately triggered at a 50-μs interval to produce dual-wavelength photoacoustic A-line pairs with a spatial interval of 0.1 μm. Statistical, spectral, and correlation analysis of 100 successive A-line pairs allows simultaneous quantification of C_Hb_, sO_2_, and CBF at the same spatial scale (10 μm). With 200 pairs of dual-color laser pulses, an ultrasonic pulse (energy: 1 μJ) is fired for pulse-echo imaging. The corresponding step size of SAM is 20 μm, which is about half of its lateral resolution.

### Quantification of absolute C_Hb_

Statistical analysis of the PAM signal can reveal the Possion distribution-governed Brownian motion of hemoglobin-carrying RBCs *in vivo*^22^. Briefly, the average RBC count 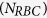 within the detection volume of PAM can be derived as:


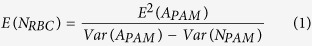


in which 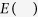 and 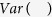 respectively denote the mean and variance operation, 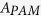 is the amplitude of the PAM signal, and 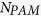 is the electronic thermal noise of our PAM system. In the present study, 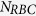 is quantified by analyzing 100 successive A-lines acquired at 532 nm. Since each RBC contains ~15 pg of hemoglobin on average^27^, the total amount of hemoglobin within the detection volume is 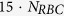 pg. Given that the lateral resolution of PAM is 2.7 μm and the 1/*e* penetration of 532-nm light in rodent blood is 46 μm^29^, the detection volume of our system is 263 μm^3^. Thus, the C_Hb_ (g/L) can be estimated as:


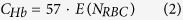


To examine the accuracy of this method *in vitro*, we prepared 10 samples with C_Hb_ evenly distributed over the range of 15–150 g/L using fresh defibrinated bovine blood (910–100, Quad Five). As shown in Supplementary Fig. S7, the PAM-measured C_Hb_ values agreed with the preset concentrations (linearity: 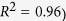 but became inaccurate when the C_Hb_ was diluted to below 30 g/L. This inaccuracy is likely due to the insufficient signal-to-noise ratio of PAM under the severe and non-physiological hemodilution.

### Vessel orientation-dependent Correction of CBF

To derive the total CBF from its transverse component measured by the correlation analysis, we have developed a three-step procedure. First, the vascular skeleton is extracted from the two-dimensional maximum amplitude-projected PAM image using a built-in function of MATLAB (bwmorph) and transformed into 3D by the incorporation of corresponding depth information. Then, the vessel axis is estimated by the local slope of the vascular skeleton on a pixel basis. Finally, by computing the angle (α) formed by the vessel axis and the transverse plane (Supplementary Fig. S8), we can derive the total CBF as:


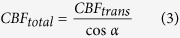


in which 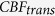 is the transverse component of the total flow speed.

### Animal preparation

We used C57BL/6 mice (4–6 weeks old, Jackson Laboratory) for the *in vivo* studies. Throughout the experiments, the mice were maintained under anesthesia with 1.0–1.5% vaporized isoflurane and the body temperature was kept at 37 °C using a temperature-controlled heating pad (Cole-Parmer, EW-89802-52; Omega, SRFG-303/10). All experimental procedures were carried out in conformity with the laboratory animal protocol approved by the Animal Care and Use Committee at the University of Virginia.

## Additional Information

**How to cite this article**: Ning, B. *et al.* Ultrasound-aided Multi-parametric Photoacoustic Microscopy of the Mouse Brain. *Sci. Rep.* **5**, 18775; doi: 10.1038/srep18775 (2015).

## Supplementary Material

Supplementary Information

Supplementary Figure S1

Supplementary Figure S2

Supplementary Figure S3

Supplementary Figure S4

Supplementary Figure S5

Supplementary Figure S6
