# Supplementary material for: Ultrasound-aided Multi-parametric Photoacoustic Microscopy of the Mouse Brain
Source: Sci Rep. 2015 Dec 21;5:18775. doi: 10.1038/srep18775 (PMC4685318; doi:10.1038/srep18775)
Supplement: Supplementary Information [file srep18775-s1.pdf]

Supplementary Information

Ultrasound-aided Multi-parametric Photoacoustic Microscopy of the Mouse Brain

Bo Ning[1], Naidi Sun[1], Rui Cao[1], Ruimin Chen[2], K. Kirk Shung[2], John A. Hossack[1], Jin-Moo Lee[3], Qifa Zhou[2,*], and Song Hu[1,*]

[1]Department of Biomedical Engineering, University of Virginia, Charlottesville, VA 22908

[2]Department of Biomedical Engineering, University of Southern California, Los Angeles, CA 90089

[3]Department of Neurology, Washington University School of Medicine, St. Louis, MO 63110

* Corresponding authors: Song Hu (photoacoustic microscopy) songhu@virginia.edu

Qifa Zhou (ultrasonic transducer) qifazhou@usc.edu

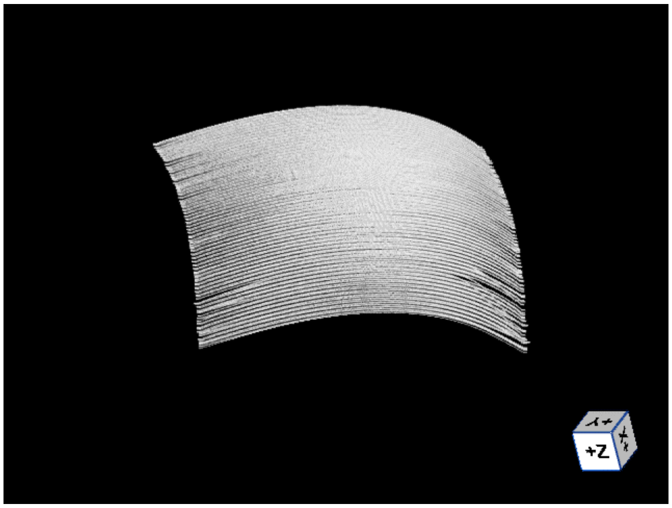

Supplementary Figure S1 | A movie showing the 3D surface contour of the plastic ball extracted by SAM.

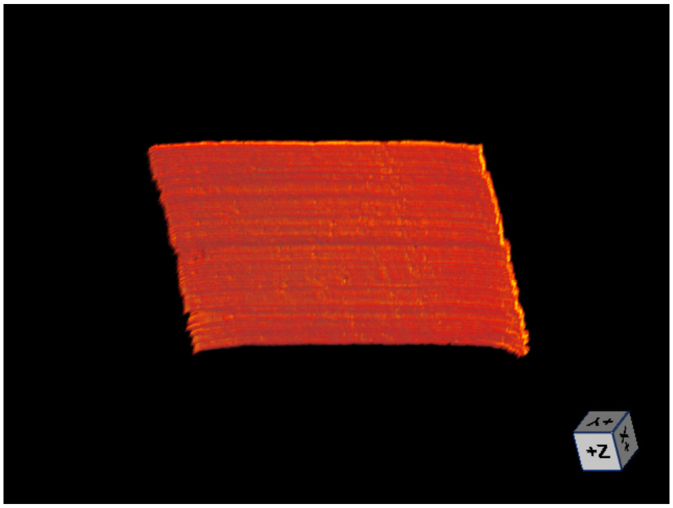

Supplementary Figure S2 | A movie showing the 3D rendering of the ball surface imaged by PAM with contour scan.

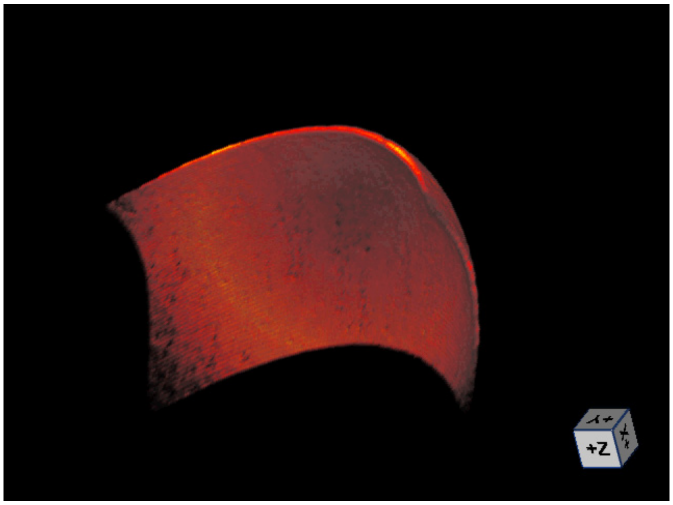

Supplementary Figure S3 | A movie showing the 3D rendering of the ball surface imaged by PAM without contour scan.

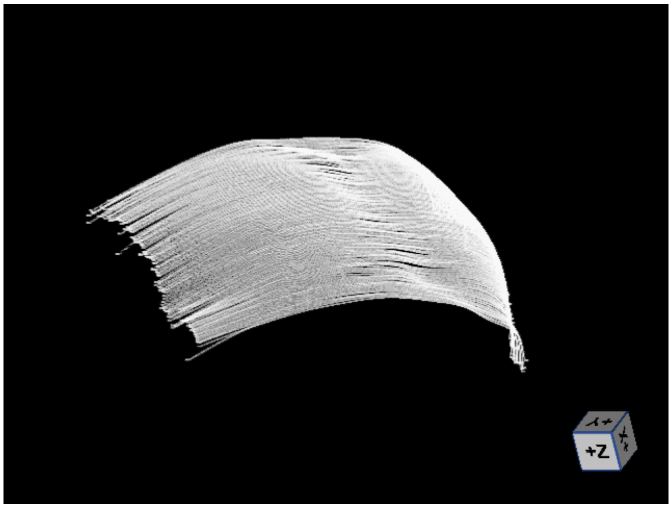

Supplementary Figure S4 | A movie showing the 3D surface contour of the mouse skull extracted by SAM.

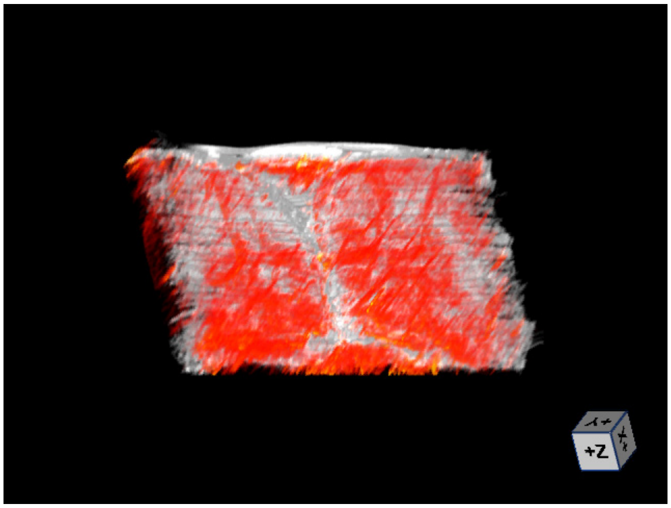

Supplementary Figure S5 | A movie showing the 3D rendering of the mouse brain simultaneously imaged by SAM (gray) and PAM (hot) with contour scan.

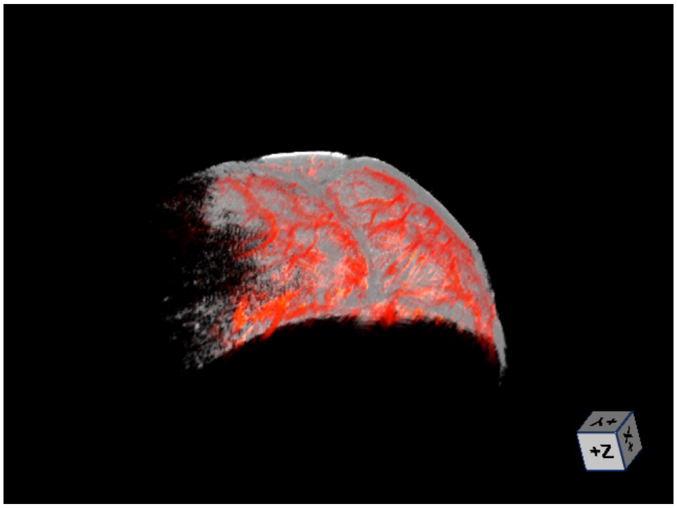

Supplementary Figure S6 | A movie showing the 3D rendering of the mouse brain simultaneously imaged by SAM (gray) and PAM (hot) without contour scan.

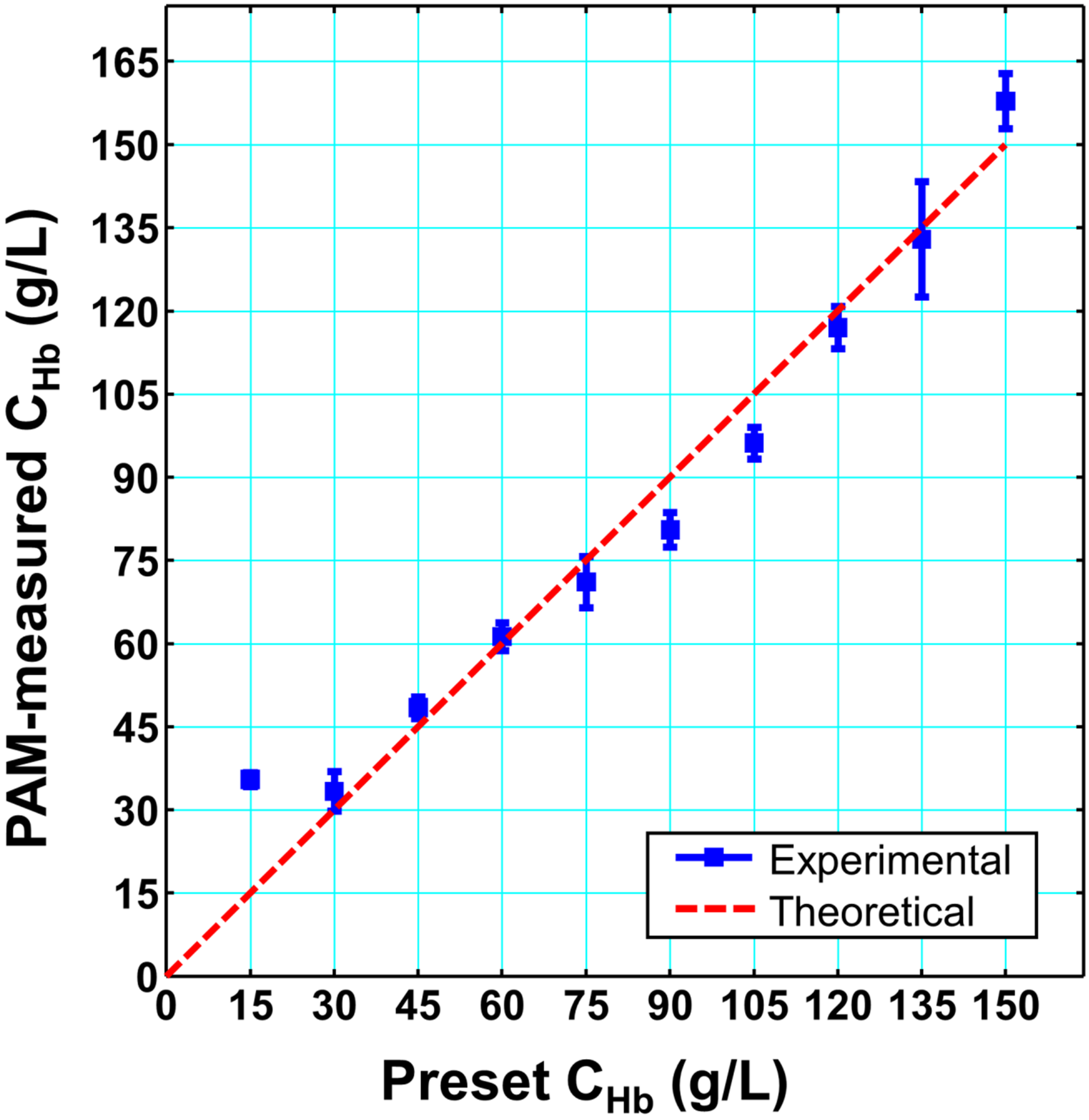

Supplementary Figure S7 | Validation of PAM-based C_{Hb} quantification using defibrinated bovine blood *in vitro*.

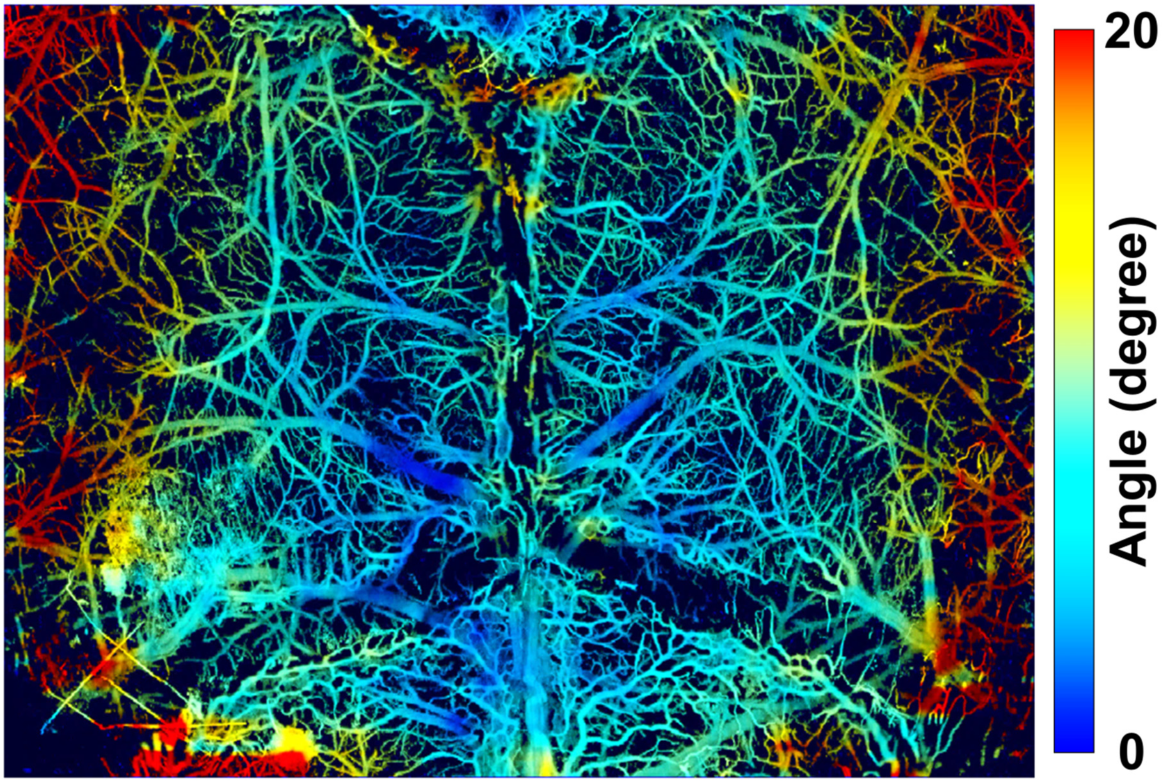

Supplementary Figure S8 | Orientation of the brain vasculature determined by the ultrasound-aided contour PAM. The angle (α) is formed by the vessel axis and the transverse plane.
